# Self-rated health, mental wellbeing, nutrition habits, and their association with morbidity of ischemic heart disease

**DOI:** 10.3389/fpubh.2025.1722066

**Published:** 2026-01-12

**Authors:** Giedrė Aukstakalniene, Dalia Luksiene, Abdonas Tamosiunas, Lolita Sileikiene, Vaiva Lesauskaite

**Affiliations:** Institute of Cardiology of Lithuanian University of Health Sciences, Kaunas, Lithuania

**Keywords:** ischemic heart disease, mental wellbeing, nutrition habits, physical activity, self-rated health, sleep hours

## Abstract

**Background:**

Lifestyle factors - such as dietary habits, physical activity, smoking, and sleep quality - are modifiable determinants that not only directly affect cardiovascular risk but also shape an individual’s self-rated health and mental wellbeing. However, the interrelationships among these domains remain inadequately understood. The aim of this study is a comprehensive assessment of complex factors - self-rated health, mental wellbeing, and lifestyle factors and their association with morbidity of ischemic heart disease (IHD).

**Materials and methods:**

This epidemiological health survey of the study “Chronic diseases and their risk factors in the adult population” was performed during 2023–2024 in Kaunas city (Lithuania) following the methodology of the WHO MONICA study. A random sample of Kaunas inhabitants aged 25–69 years, stratified by sex and age, was randomly selected from the Lithuanian population register. The 3,426 individuals were screened. The associations of self-rated health, mental wellbeing status, and lifestyle habits with the IHD were investigated using binary logistic regression analysis. An exploratory factor analysis with orthogonal varimax rotation was conducted to examine the factor structure of the food intake data.

**Results:**

This study highlights the significant association of good self-rated health and mental wellbeing status with a lower odds of IHD, even after adjusting for main covariates (sex, age, education, marital status, metabolic syndrome, and its components) (OR = 0.692, 95% CI: 0.22–0.93, *p* = 0.013). Furthermore, specific lifestyle habits - including regular physical activity (OR = 1.661, 95% CI: 1.35–2.04, *p* < 0.001), sufficient sleep (OR = 1.115, 95% CI: 1.03–1.21, *p* = 0.006), and healthy nutrition habits rich in fruits, vegetables, and fish (OR = 1.463, 95% CI: 1.24–1.73, *p* < 0.001) - were positively associated with better self-rated health and mental wellbeing. However, more frequent consumption of meat products and potatoes reduced the odds of good self-rated health and mental wellbeing (OR = 0.831, 95% CI: 0.70–0.99, *p* = 0.04).

**Conclusion:**

These findings underscore the importance of promoting healthy lifestyle behaviours and mental wellbeing as part of comprehensive cardiovascular disease prevention strategies.

## Introduction

Cardiovascular diseases (CVD) remain a leading cause of morbidity and mortality worldwide, contributing significantly to the global burden of non-communicable diseases (1). Despite advancements in the early diagnosis and treatment of CVD during the last decades, Lithuania has had the highest mortality rate from CVD among all causes of death. In 2024, according to the data from the Health Information Centre of the Institute of Hygiene (Lithuania), more than half (50.8%) of all deaths occurred due to diseases of the circulatory system (2). Ischemic heart disease (IHD) mortality has fallen, although it remains the leading cause of death in 2021. In Lithuania, IHD was the leading cause of death, accounting for almost one-third of all deaths (3). In 2024, according to data from the Health Information Centre of the Institute of Hygiene (Lithuania), the leading cause of death for both women and men was diseases of the circulatory system, accounting for 56.8% of deaths among women and 44.6% among men. Among deaths due to circulatory system diseases, 59.2% of women and 60.4% of men died from IHD (2).

Notably, despite the newest therapeutic modalities, the prevalence of the most common of the cardiovascular diseases—IHD continues to be influenced by a constellation of behavioral, psychological, and perceptual health determinants. Among these, self-rated health, psychological wellbeing, and lifestyle factors have emerged as important complex contributors to cardiovascular risk profiles (4–7).

Recent scientific literature shows a shift from defining wellbeing merely as the absence of disease toward a more holistic understanding that encompasses mental, physical, and social health (8–11). This broader perspective aligns with the ‘One Health’ approach and has influenced public health initiatives, including the development of leading health indicators that are now internationally recognized tools for assessing wellbeing. One of the health monitoring indicators recommended by the European Union Commission and the WHO is self-reported health (12). This measure offers participants the opportunity to assess their own health from a personal perspective. Also, it is one of the most used measures of perceived health status that encompasses a person’s biological, mental, social, and functional aspects (13). Another health monitoring indicator is the World Health Organisation’s Five-item wellbeing index (WHO-5). It is a widely recognized and utilised short self-reported screening tool designed to assess population mental wellbeing, public health, and overall quality of life (14). In various studies, the WHO-5 score has shown a correlation with other psychological parameters, including depression, anxiety, stress, and overall mental health (15).

Lifestyle factors - such as dietary habits, physical activity, smoking, and sleep quality - are modifiable determinants that not only directly affect cardiovascular risk but also shape an individual’s self-rated health and mental wellbeing (16–18). For instance, regular physical activity has been shown to improve both mood and subjective health ratings, while poor sleep is associated with diminished wellbeing and elevated cardiovascular risk (19–21). However, the interrelationships among these domains - how lifestyle behaviours influence self-rated health and mental wellbeing, and in turn, how these factors collectively affect IHD morbidity - remain inadequately understood.

The aim of this study is a comprehensive assessment of complex factors - self-rated health, mental wellbeing, and lifestyle factors and their association with morbidity of IHD. A particular focus is placed on identifying which lifestyle factors contribute to better self-rated health and mental wellbeing, which ultimately could reduce the risk of IHD.

## Materials and methods

### Study population

This epidemiological health survey of the study “Chronic Diseases and their Risk Factors in the Adult Population” (Study) was performed in Kaunas city (Lithuania) following the methodology of the WHO programme Multinational Monitoring of Trends and Determinants in Cardiovascular Disease (MONICA) study (22, 23). A random sample of Kaunas males and females aged 25–69 years, stratified by sex and age, was randomly selected from the Lithuanian population register (*N* = 6,000). The call for participants started on February 1, 2020, but due to the COVID-19 pandemic, the Study was suspended on March 15, 2020, and in March 2023, the study was resumed and conducted until June 21, 2024. The 3,426 individuals (1,523 males and 1,903 females) were screened. The response rate was 57.1%. The Study was approved by the Kaunas Regional Ethics Committee (Lithuania) (Nr. BE-2-49; 2018-06-05). All participants provided written informed consent. Inclusion criteria: all selected people are invited. No exclusion criteria were applied.

### Variables determined using the questionnaire

During the health examination, trained interviewers gathered sociodemographic and health-related data using a structured questionnaire, which included items on age, sex, marital status, and educational level. Marital status was dichotomized as married (married, cohabiting) and single (single, divorced, separated, and widowed). Education was measured by six education levels: primary, incomplete secondary, secondary, vocational, college, and university. The responders with primary, incomplete secondary, and vocational education were considered as having secondary and lower education.

Health-related variables included self-rated health evaluation, WHO-5, and lifestyle factors (smoking habits, physical activity and nutrition habits, and sleeping hours). Self-rated health has been evaluated according to the answers of the health survey participants to the question “How do you rate your current health status?” Answers: 1 - very good; 2 - good; 3 - average; 4 - poor; 5 - very poor. The responders were categorized into three groups according to their self-related health: very good and good; average; poor and very poor.

Mental wellbeing status was evaluated according to the WHO-5 questionnaire, which was translated into Lithuanian in collaboration with the WHO Collaborating Centre in Mental Health Psychiatric Research Unit, and the Lithuanian version was officially approved in 1999 (24). The calculation methodology was specified in the questionnaire. The answers to each of the five statements which are closest to how responders have been feeling over the last 2 weeks. Each of the five questions has a score from 0 (At no time) to 5 (All of the time). The raw score was calculated by totalling the scores on each of the five questions. So, the raw score ranges from zero to 25. To get a percentage score, the raw score is multiplied by four. A higher score indicates better wellbeing. The total ranges from 0 to 100%, with a median of 64%. Following this methodology, we divided the subjects into two groups based on the median score: poor mental wellbeing status (≤64%) and good mental wellbeing status (>64%).

For assessing smoking habits, respondents were asked: “Do you smoke at the moment?” Answers: 1- yes, daily at least one cigarette a day on average; 2 - former (No, I smoked in the past but I stopped), 3 - no, I have never smoked. Smoking was categorized as never, former, and current regular smoking (smoking at least 1 cigarette per day each day). To assess physical activity during leisure time, respondents were asked: “How many hours per week during your leisure time do you spend separately in the autumn-winter and spring–summer seasons? Answers (in hours): walking, gardening, house maintenance, and other physical activities. Physical activity was calculated by summarizing time spent per week separately in the autumn-winter and spring–summer seasons for activities such as walking, gardening, house maintenance, and other physical activities. The participants were divided into three equal groups (tertiles) according to the mean length of time spent per week on physical activities. The 1st tertile maximal cut-off was 5 h per week (mean 2.83 (SD = 1.53)), the 2nd tertile maximal cut-off was 9.5 h per week (mean 7.25 (SD = 1.24)), and the 3rd tertile maximal cut-off was 42 h per week (mean 15.3 (SD = 5.85)). Sleeping hours have been evaluated according to the answers of the health survey participants to the question “How many hours do you sleep per night on average?”

Nutrition habits were evaluated using a food frequency questionnaire. 30 food groups were included in the food frequency questionnaire: porridges and cereals, pasta, legumes (beans, lentils, peas), whole grain bread, potatoes (boiled and fried), dairy products (cheese, curd cheese, sour cream, milk), meat (chicken, beef, veal, turkey, beef, pork, lamb, goose), smoked meat products and sausage, fish, eggs, fresh vegetables (in summer and autumn; in winter and spring), boiled and canned vegetables, fresh fruits and berries (in summer and autumn; in winter and spring), candies and chocolate, cakes, fast food (kebabs, hamburgers, etc.), snacks (chips, popcorns), sweet drinks (Coca-Cola, Pepsi). Possible responses for all food groups were: 1 - rarely or never; 2 - 1–3 times a month, 3 - 1 time a week, 4 - few times a week, 5 - daily; 6 - few times a day. Higher values denote more frequent use of the current food. Mean values of the use of fresh vegetables, fresh fruit, and berries in summer and autumn, and in winter and spring were calculated, and 28 food groups in the final analysis were included.

Factor analysis was employed to reduce the number of food items. Exploratory factor analysis with orthogonal varimax rotation was conducted to explore the factor structure of food intake data. Eigenvalues and directions of factor loadings explaining variance were analysed. The Kaiser–Meyer–Olkin (KMO) test was used to measure sample adequacy. The Kaiser rule (eigenvalue greater than one) was used to determine the number of factors to be indicated (25). We used the varimax method to obtain orthogonal factors. A six-factor solution was indicated using this method. Using the varimax rotation method, each factor tends to have either large or small loadings of any particular variable. A varimax solution yields results that make it as easy as possible to identify each variable with a single factor (25). Direct oblimin rotation is a non-orthogonal solution method that is one, in which factors are allowed to be correlated. This can result in higher eigenvalues but diminished interpretability of the factors (25). In our case, the purpose of the factor analysis was to explore the factor structure of the nutrition habits without any prior suggestion of how many factors there are or whether they are correlated. KMO resulted in a measure of sampling adequacy of 0.783 and indicated the moderate appropriateness to proceed with factor analysis (*p* < 0.001). A six-factor solution, which accounted for 43.0% of the total variance, was indicated. Table 1 presents factors and factor loadings for the dietary variables of the epidemiological health survey of the study “Chronic Diseases and their Risk Factors in the Adult Population” (Kaunas health survey in the 25–69 years old population). Factor loadings of <0.4 were excluded from Table 1 for simplicity. Each food factor group was constructed as a dichotomous dependent variable by dividing factor scores into groups (1 - more frequent than average consumption of food items in the particular food group; 0 - less frequent than average consumption of food items). Table 2 presents the proportions of more frequent than average consumption of food items in each factor food group.

**Table 1:** Factors and factor loadings for the dietary variables of the responders aged 25–69 years of the Kaunas health survey (2023–2024).

Type of food	Factor 1st	Factor 2nd	Factor 3rd	Factor 4th	Factor 5th	Factor 6th
	Fast food	Meat products, potatoes	Vegetables, fruit, fish	Dairy products, eggs	Sweets	Cereals, pasta
Snacks (chips, popcorns)	0.736					
Fast food (kebabs, hamburgers, etc.)	0.736					
Sweet drinks (Coca-Cola, Pepsi)	0.658					
Potatoes (boiled)		0.727				
Pork, greens, lamb		0.586				
Sausage		0.527				
Smoked meat products		0.496				
Potatoes (fried)		0.487				
Fresh vegetables			0.631			
Boiled and canned vegetables			0.575			
Legumes (beans, lentils, peas)			0.553			
Fresh fruits and berries			0.533			
Fish			0.433			
Curd cheese				0.661		
Yoghurt				0.650		
Fermented cheese				0.521		
Eggs				0.458		
Milk				0.412		
Cakes					0.790	
Candies, chocolates					0.780	
Cereals						0.593
Porridges (buckwheat, rice, oats)						0.514
Pasta						0.493

Factor loadings of <0.4 were excluded from the table for simplicity.

**Table 2:** Baseline characteristics of responders of the Kaunas health survey (2023–2024).

Variables	Value
Number of responders, *N*	3,426
Age, years, mean (SD)	49.1 (11.24)
Male, % (*n*)	44.5 (1523)
Education, % (*n*)	
Primary + Vocational + Secondary	31.2 (1066)
College	19.1 (653)
University	49.8 (1703)
Marital status, % (*n*)	
Single + Divorced + Widowed	27.5 (940)
Married + Cohabiting	72.5 (2480)
Self-rated health, % (*n*)	
Very good + good	60.9 (2086)
Average	35.5 (1216)
Poor	3.6 (124)
WHO-5 mental wellbeing index, mean (SD)	65.31 (17.88)
WHO-5 mental wellbeing index groups	
Good mental wellbeing	51.10 (1622)
Poor mental wellbeing	48.9 (1552)
IHD, % (*n*)	11.3 (387)
Metabolic syndrome, % (*n*)	31.9 (1087)
Metabolic syndrome components, % (*n*)	
Elevated arterial blood pressure (≥130/85 mm/Hg), % (*n*)	61.2 (2096)
Increased waist circumference, (men ≥ 102 cm, women ≥ 88 cm), % (*n*)	43.4 (1485)
HDL cholesterol (men < 1.0 mmol/L, women < 1.3 mmol/L), % (*n*)	39.5 (1349)
Triglycerides ≥ 1.7 mmol/L, %	25.9 (885)
Fasting glucose ≥ 6.1 mmol/L, %	16.8 (573)
Body mass index, kg/m^2^, mean (SD)	27.3 (5.29)
Body mass index groups % (*n*)	
Normal	36.5 (1250)
Overweight	37.5 (1,284)
Obesity	26.0 (890)
Smokers, % (*n*)	19.5 (666)
Physical activity in leisure time, hours/week, mean (SD)	8.63 (7.05)
Physical activity in leisure time tertiles, mean (SD)	
1st tertile	2.83 (1.53)
2nd tertile	7.25 (1.24)
3rd tertile	15.3 (5.85)
Nutrition habits	
More frequent^*^ fast-food consumption, % (*n*)	44.1 (1,508)
More frequent consumption of meat products and potatoes, % (*n*)	54.0 (1,846)
More frequent fresh vegetables, fruit, and fish consumption, % (*n*)	52.7 (1,802)
More frequent dairy product consumption, % (*n*)	52.8 (1,806)
More frequent sweets consumption, % (*n*)	52.0 (1,779)
More frequent porridge, cereals, and pasta consumption, % (*n*)	48.4 (1,656)
Sleep hours per night, mean (SD)	7.15 (1.05)

SD, standard deviation; WHO, World Health Organization; HDL, high-density lipoprotein.

* The proportions of more frequent than average consumption of food items in each factor food group.

### Measurements

The body weight was measured with a calibrated medical scale, without shoes or heavy clothes. Weight values were recorded to the nearest 100 g. The height of participants (without shoes) was measured with an accuracy of 1 cm, using a stadiometer. Body mass index (BMI) was calculated using the following formula: BMI = body mass (kg)/height^2^ (m^2^). The health survey participants were divided into groups: group with normal weight (BMI 18.5–24.99 kg/m^2^), overweight (BMI 25.0–29.99 kg/m^2^), and obese (BMI ≥ 30.0 kg/m^2^). Insufficient weight was defined as BMI < 18.5 kg/m^2^.

Blood pressure was measured two times with an oscillometric device (Omron M5-1) after at least 5 min. of rest in a seated position, and mean values of systolic and diastolic blood pressure were taken.

Laboratory tests included measurements of triglyceride, high-density lipoprotein (HDL) cholesterol, and glucose after fasting overnight for at least 8 h. The serum was collected from the patients using venipuncture tubes to ensure that the sample was free from haemolysis. A 200 μL sample of the serum was used for each test. Enzymatic colorimetric analysis was performed using the Selectra PRO XS biochemistry analyzer from EliTech Group (The Netherlands). The reagents from EliTech Group B. V. (The Netherlands) were used for high-density lipoprotein cholesterol and triglycerides. All the analytes were detected directly by measuring the sample’s absorption at 500 nm. To ensure accuracy, Randox calibrators for cholesterol were used. Calibration was performed according to the instrument and kit manufacturer’s recommendations. HDL-cholesterol calibrators (Randox, Crumlin, UK) and Clinical chemistry calibration serum level 3 (Randox, Crumlin, UK) were used. For day-to-day internal quality control assessment, Lipid Control Levels 1, 2, and 3 (Randox, Crumlin, UK) were used. The precision and accuracy were ensured by comparing the quality control sample results to expected values. The method used for measuring plasma glucose was GLUCOSE OXIDASE-PAP/End Point Enzymatic PAP using the Selectra PRO XS biochemistry analyzer.

The diagnostic criteria for metabolic syndrome by the Third Report of the National Cholesterol Education Program Adult Treatment Panel III (NCEP-ATP III) definition are the presence of three or more of the following risk determinants: (1) increased waist circumference (≥102 cm for men and ≥88 cm for women); (2) elevated triglycerides (≥1.7 mmol/L); (3) low HDL cholesterol (<1.0 mmol/L in men and <1.3 mmol/L in women); (4) arterial hypertension (≥130/85 mmHg); and (5) fasting glucose (≥6.1 mmol/L) (26).

### Identification of IHD

To assess myocardial infarction history, respondents were asked: “Have you ever had a myocardial infarction?” Answers: 1 - no, 2 - yes. If “yes,” then in which hospital and in what year were you treated? An electrocardiogram (ECG) was recorded for each responder and assessed by Minnesota codes. The epidemiological criteria for the identification of IHD at the time of the health check according to the priority are: (1) exposure to myocardial infarction and/or ischemic changes on the ECG, as assessed by Minnesota codes 1–1, 1–2 (27); (2) exertional angina pectoris identified using G. Rose questionnaire (28); (3) ischemic ECG changes assessed using the following Minnesota codes: 1–3, 4–1, 4–2, 4–3, 5–1, 5–2, 5–3, 6–1, 6–2, 7–1, 8–3.

### Statistical analysis

All statistical analyses in this investigation were executed using IBM SPSS Statistics (Version 29.0) (IBM Corp. Released 2023. IBM SPSS Statistics for Windows, Version 29.0. Armonk, NY, USA). All continuous variables included in the analysis were tested for normality (by skewness and kurtosis values) and homogeneity of variance (by Levene’s test). The differences in means of variables between groups were assessed using *T*-test and ANOVA test for variables that were normally distributed and met the conditions of homogeneity of variance. A chi-squared test and *z*-test were used to assess the differences in categorical variables. Significance levels have been adjusted by the Bonferroni correction for multiple comparisons; *p* < 0.05 values were considered statistically significant. The associations of self-rated health, mental wellbeing status, and lifestyle habits with the IHD, and lifestyle habits with self-rated health and mental wellbeing status were investigated using binary logistic regression analysis. The odds ratios (OR) with 95% confidence interval (CI) were computed.

For binary logistic regression analysis of associations of self-rated health, mental wellbeing status, and lifestyle habits with IHD (Table 3): the dependent variable was IHD. Independent variables were lifestyle habits (smoking status, physical activity status, nutrition habits, sleep hours) and 3 groups of self-rated health, mental wellbeing status (poor and average self-rated health and poor mental wellbeing status; good self-rated health or good mental wellbeing status; good self-rated health and good mental wellbeing status), covariates: sex, age, education, marital status, metabolic syndrome, and its components.

**Table 3:** Associations of self-rated health, mental wellbeing status, and lifestyle habits with ischaemic heart disease among 25–69-year-old population.

Variables	Model 1			Model 2			Model 3^*^		
	OR	95% CI	*p*	OR	95% CI	*p*	OR	95% CI	*p*
Self-rated health and mental wellbeing status									
Poor and average self-rated health + poor mental wellbeing status	1			1			1		
Good self-rated health or good mental wellbeing status	0.605	0.46–0.79	**<0.001**	0.737	0.56–0.97	**0.030**	0.744	0.56–0.98	**0.038**
Good self-rated health + good mental wellbeing status	0.526	0.40–0.70	**<0.001**	0.692	0.22–0.93	**0.013**	0.716	0.53–0.97	**0.028**
Smoking habits (non-smokers vs. smokers)	0.835	0.63–1.10	0.205	–			0.816	0.59–1.12	0.205
Physical activity in leisure time									
1st tertile	1			–			1		
2nd tertile	1.106	0.85–1.44	0.452	–			1.048	0.79–1.39	0.748
3rd tertile	1.118	0.86–1.447	0.400	–			1.041	0.78–1.39	0.785
Nutrition habits (No vs. Yes)									
More frequent fast-food consumption	0.678	0.54–0.85	**0.001**	–			1.060	0.81–1.39	0.678
More frequent consumption of meat products and potatoes	1.383	1.11–1.72	**0.003**	–			1.308	1.02–1.68	**0.037**
More frequent fresh vegetables, fruits, and fish consumption	0.932	0.75–1.15	0.517	–			0.987	0.78–1.25	0.916
More frequent dairy product consumption	1.080	0.87–1.34	0.476	–			0.966	0.77–1.22	0.767
More frequent consumption of sweets	0.865	0.70–1.07	0.180	–			0.873	0.69–1.10	0.255
More frequent porridge, cereals, and pasta consumption	0.894	0.72–1.11	0.302	–			0.975	0.77–1.23	0.833
Sleep hours per night, per 1 h	0.930	0.84–1.04	0.183	–			0.955	0.86–1.07	0.411

Model 1 simple binary logistic regression for each independent variable, presented in Table 5. The dependent variable was IHD.

Model 2: multivariable binary logistic regression analysis. The dependent variable was IHD. Independent variables: 3 groups of self-rated health, mental wellbeing status (poor and average self-rated health and poor mental wellbeing status; good self-rated health or good mental wellbeing status; good self-rated health and good mental wellbeing status). Covariates: sex, age, education, marital status, metabolic syndrome, and its components.

Model 3: multivariable binary logistic regression analysis. The dependent variable was IHD. Independent variables: lifestyle habits (smoking status, physical activity status, nutrition habits, sleep hours) and 3 groups of self-rated health, mental wellbeing status (poor and average self-rated health and poor mental wellbeing status; good self-rated health or good mental wellbeing status; good self-rated health and good mental wellbeing status). Covariates: sex, age, education, marital status, metabolic syndrome, and its components.

* The main differences between Model 2 and Model 3 were the inclusion of different independent variables. Independent variables in Model 2 were: three groups of self-rated health, mental wellbeing status: poor and average self-rated health and poor mental wellbeing status; good self-rated health or good mental wellbeing status; good self-rated health and good mental wellbeing status. Independent variables in Model 3 were the same three groups of self-rated health, mental wellbeing status, plus lifestyle habits (smoking status, physical activity status, nutrition habits, sleep hours).

OR, odds ratio adjusted for variables in the models; CI, confidence interval.Bold typeface indicates significance.

For binary logistic regression analysis of associations of lifestyle habits with self-rated health and mental wellbeing status (Table 4), as a dependent composite variable, self-rated health and mental wellbeing status was divided into two groups: 0 - Poor and average self-rated health + poor mental wellbeing status; and 1 - good self-rated health and/or good mental wellbeing, because two groups were combined (Good self-rated health or good mental wellbeing status plus good self-rated health + good mental wellbeing status). Independent variables were lifestyle habits (smoking status, physical activity status, nutrition habits, sleep hours), covariates: sex, age, education, and marital status.

**Table 4:** Associations of lifestyle habits with good self-rated health and/or good mental wellbeing status among 25–69-year-old population.

Exposure	Model 1			Model 2		
	OR	95% CI	*p*	OR	95% CI	*p*
Smoking habits (non-smokers vs. smokers)	0.866	0.71–1.05	0.149	0.915	0.74–1.13	0.12
Physical activity in leisure time						
1st tertile	1			1		
2nd tertile	1.168	0.97–1.41	0.106	1.155	0.95–1.41	0.150
3rd tertile	1.664	1.36–2.03	**<0.001**	1.661	1.35–2.04	**<0.001**
Nutrition habits (No vs. Yes)						
More frequent fast-food consumption	1.535	1.30–1.81	**<0.001**	1.073	0.88–1.31	0.479
More frequent consumption of meat products and potatoes	0.776	0.66–0.91	**0.002**	0.831	0.70–0.99	**0.040**
More frequent fresh vegetables, fruits, and fish consumption	1.508	1.29–1.77	**<0.001**	1.463	1.24–1.73	**<0.001**
More frequent dairy product consumption	1.023	0.87–1.20	0.781	1.027	0.87–1.21	0.749
More frequent consumption of sweets	0.974	0.83–1.14	0.747	0.933	0.79–1.10	0.415
More frequent porridge, cereals, and pasta consumption	1.079	0.92–1.27	0.346	0.989	0.84–1.17	0.899
Sleep hours per night, per 1 h	1.136	1.05–1.23	**0.001**	1.115	1.03–1.21	**0.006**

Model 1: simple binary logistic regression for each independent variable, presented in Table 3. The dependent variable: good self-rated health and/or good mental wellbeing.

Model 2: multivariable binary logistic regression analysis. The dependent variable was good self-rated health and/or good mental wellbeing. Independent variables: lifestyle habits (smoking status, physical activity status, nutrition habits, sleep hours). Covariates: sex, age, education, marital status.

OR, odds ratio adjusted for variables according to models; CI, confidence interval.Bold typeface indicates significance.

## Results

### Characteristics of study group

The baseline characteristics of the respondents in the Kaunas health survey in the 25–69 age population are presented in Table 2. This health survey included 3,426 participants, of whom 44.5% were male. The mean age (SD) of participants was 49.1 (11.24) years. Approximately half of the participants had a university education, and 72.5% were married or cohabiting. More than half of the respondents (60.9%) reported their health as very good or good, while 35.5% rated their health as average, and only 3.6% indicated their health as poor. More than 50% of respondents had a good WHO-5 mental wellbeing index. IHD was diagnosed in 11.3% of respondents, while metabolic syndrome was identified in 31.9%. The most common component of metabolic syndrome was elevated arterial blood pressure (61.2%), while the least common was high fasting glucose levels (16.8%). Obesity was found in 26.0% of respondents, and 19.5% were smokers. The mean (SD) physical activity during leisure time was 8.63 (7.05) hours per week, and the mean sleep duration per night was 7.15 (1.05) hours.

### Findings

The relationship between self-rated health and mental wellbeing as measured by the WHO-5 mental wellbeing index shows that respondents who rated their health as “Very good and good” (*n* = 1,922) had the highest mean of mental wellbeing index score of 69.55 (SD = 16.0). Respondents who rated their health as “Average” (*n* = 1,140) had a lower mean mental wellbeing index score of 59.95 (SD = 17.9), and the respondents who rated their health as “Poor” (Poor + very poor) (*n* = 112) had the lowest mean mental wellbeing index score of 47.21 (SD = 21.5). The *p*-value < 0.001 suggests a statistically significant difference in mental wellbeing scores across the self-rated health categories. Thus, the respondents were divided into three groups based on their self-rated health and WHO-5 mental wellbeing index: the first group—responders who had poor and average self-rated health plus poor mental wellbeing status (*n* = 826); the second group - responders who had good self-rated health or good mental wellbeing status (*n* = 1,222), and third group—responders who had good self-rated health plus good mental wellbeing status (*n* = 1,126). The characteristics of three groups of responders based on their self-rated health and WHO-5 mental wellbeing index are presented in Table 5. Frequency of some sociodemographic characteristics (age, education level, marital status) and lifestyle habits such as BMI, physical inactivity (1st tertile), nutrition habits (more frequent than average fast-food, meat products, and potatoes consumption, fresh vegetables, fruit, and fish consumption), and sleep hours per night were significantly different as compared with responders with poor and average self-rated health, plus poor mental wellbeing status.

**Table 5:** Characteristics of three groups of responders based on their self-rated health and WHO-5 mental wellbeing index (The Kaunas health survey (2023–2024)).

Variables	Responders with poor and average self-rated health, plus poor mental wellbeing status	Responders with good self-rated health or good mental wellbeing status	Responders with good self-rated health plus good mental wellbeing status	*p*
Number of responders, *N*	*n* = 826	*n* = 1,222	*n* = 1,126	
Age, years, mean (SD)	52.6 (10.9)	48.9 (11.0)^a^	47.5 (10.9)^a,b^	<0.001
Male, % (*n*)	43.2	42.2	45.3	0.317
Education, % (*n*)				
Primary + Vocational + Secondary	37.5	29.3^a^	29.2^a^	<0.001
College	19.5	19.1	17.9	
University	43.0	51.6^a^	52.9^a^	
Marital status, % (*n*)				
Single + Divorced + Widowed	67.1	75.0^a^	74.4^a^	<0.001
Married + Cohabiting	32.9	25.0^a^	25.6^a^	
IHD, % (*n*)	15.5	10.0^a^	8.8^a^	<0.001
Body mass index groups % (*n*)				
Normal	30.8	37.9^a^	38.5^a^	<0.001
Overweight	33.9	38.5	39.2^a^	
Obesity	35.3	23.6^a^	22.3^a^	
Smokers, % (*n*)	21.2	19.6	18.1	0.242
Physical activity in leisure time tertiles, mean (SD)				
1st tertile	2.6 (1.6)	2.9 (1.5)^a^	3.0 (1.5)^a^	0.002
2nd tertile	7.2 (1.3)	7.2 (1.2)	7.3 (1.2)	0.315
3rd tertile	15.3 (6.6)	15.8 (6.4)	15.2 (6.2)	0.482
Nutrition habits				
More frequent^*^ fast-food consumption, % (*n*)	36.7	45.7^a^	48.6^a^	<0.001
More frequent consumption of meat products and potatoes, % (*n*)	58.7	54.2	50.5^a^	0.002
More frequent fresh vegetables, fruit, and fish consumption, % (*n*)	45.5	52.7^a^	58.9^a,b^	<0.001
More frequent dairy product consumption, % (*n*)	52.5	54.2	51.8	0.502
More frequent sweets consumption, % (*n*)	52.1	51.6	51.3	0.936
More frequent porridge, cereals, and pasta consumption, % (*n*)	46.7	46.2	51.1	0.039
Sleep hours per night, mean (SD)	7.0 (1.2)	7.1 (1.0)	7.3 (1.0)^a,b^	<0.001

SD, standard deviation; WHO, World Health Organization; HDL, high-density lipoprotein.

^*^The proportions of more frequent than average consumption of food items in each factor food group.

^a^*p* < 0.05 as compared with responders with poor and average self-rated health, plus poor mental wellbeing status.

^b^*p* < 0.05 as compared with responders with good self-rated health or good mental wellbeing status.Bold typeface indicates significance.

Table 3 presents the associations between self-rated health, mental wellbeing status, lifestyle habits, and IHD in the 25–69-year-old population. In Model 1 (simple binary logistic regression analysis) and Model 2 (multivariable binary logistic regression analysis: including covariates sex, age, education, marital status, metabolic syndrome and its components), good self-rated health plus good mental wellbeing status significantly associated with a lower odds of IHD (OR = 0.526 (95% CI 0.40–0.70) and OR = 0.692 (95% CI 0.22–0.93), respectively) compared to respondents with poor and average self-rated health plus poor mental wellbeing status. Importantly, having at least one of these factors: either good self-rated health or good mental wellbeing, also was significantly associated with a lower odds of IHD in Model 1 and Model 2 (OR = 0.605 (95% CI 0.46–0.79) and OR = 0.737 (95% CI 0.56–0.97), respectively) compared to the respondents with poor and average self-rated health plus poor mental wellbeing status. Model 3 presents the associations of lifestyle habits (smoking, physical activity, dietary habits, hours of sleep) with IHD among the population aged 25–69 years, with self-rated health and mental wellbeing (Table 5). From all analysed lifestyle factors, only more frequent consumption of meat products and potatoes was positively associated with the risk of IHD (OR = 1.308, 95% CI: 1.02–1.38). However, despite the inclusion of multiple potential IHD independent risk factors in Model 2 and Model 3, good self-rated health plus good mental wellbeing, and good self-rated health or good mental wellbeing were associated with a lower odds of IHD. For this reason, we combined these two groups based on self-rated health and mental wellbeing, as both were significantly associated with reduced odds of IHD. Therefore, in the subsequent analysis, we categorized the respondents into groups: the reference group included those with both poor self-rated health and poor mental wellbeing, the comparison group included those with good self-rated health and/or good mental wellbeing. It is important to identify which modifiable lifestyle risk factors contribute to good self-rated health and/or good mental wellbeing.

Table 4 presents associations of lifestyle habits with good self-rated health and/or good mental wellbeing status among the 25–69-year-old population. In Model 1 (simple binary logistic regression analysis) and in Model 2 (multivariable binary logistic regression analysis: including independent variables: lifestyle habits) (smoking status, physical activity status, nutrition habits, sleep hours) and covariates (sex, age, education, marital status), results indicate that lifestyle habits such as physical activity in leisure time (3rd tertile vs. 1st tertile), increased per 1-h sleep duration per night, significantly associated with a higher odds of good self-rated health and/or good mental wellbeing status in Model 1 and Model 2. Also, in both models, more frequent than average fresh vegetables, fruits, and fish consumption (3rd Factor)was associated with a higher odds of good self-rated health and/or good mental wellbeing status (respectively OR = 1.508 (95% CI 1.29–1.77) and OR = 1.463 (95% CI 1.24–1.73) On the contrary, more frequent consumption of meat products and potatoes (2nd Factor) was associated with a lower odds of good self-rated health and/or good mental wellbeing status (respectively OR = 0.776 (95% CI 0.66–0.91) and OR = 0.831 (95% CI 0.70–0.99).

## Discussion

In this cross-sectional study, we demonstrated an independent association between two indices - self-rated health and the WHO-5 wellbeing Index - and the risk of IHD in the Lithuanian urban population aged 25–69 years. We also examined the relationship between lifestyle factors and both good self-rated health and good mental wellbeing.

Self-rated health and the WHO-5 WellBeing Index are well-established indicators in epidemiological research and are considered reliable predictors of morbidity and mortality (29–32). However, the results of self-rated health assessments may vary across studies due to differences in question phrasing and response categories. In our study, self-rated health was categorized as very good, good, average, poor, or very poor. We found that over 60% of participants rated their health as very good or good, while fewer than 4% rated their health as poor. For comparison, in the U.S. NHANES survey among males and females aged 30–50 years, self-rated health was assessed using the categories: poor, fair, good, very good, and excellent (33). In that study, 68.5% of respondents reported their health as good, very good, or excellent, and only 1.6% described it as poor. Other epidemiological studies of adult populations report a wide range in the proportions of individuals rating their health as very good or good (30.3–93.3%) and as poor or very poor (4.0–34.2%), depending on participants’ age, sex, presence of chronic diseases, and other socio-demographic or health-related characteristics (30, 31, 34, 35). The results from epidemiological studies that have applied the WHO-5 are more easily comparable than self-rated health results, as the index is determined using a standardized instrument and the same cut-off points for poor mental wellbeing (14). Our study found that more than 51% of participants were assessed as having good mental wellbeing. In other studies, the prevalence of good mental wellbeing ranged from 41 to 87%, depending on factors such as age, sex, nationality, occupation, presence of chronic diseases, and other characteristics of the respondents (36–38).

Previous research indicates that poor mental wellbeing contributes to unfavourable health perceptions, while low self-rated health frequently co-occurs with psychological distress. These overlapping pathways can exert combined effects on cardiovascular morbidity (39). Therefore, the composite score was created to reflect the cumulative burden of impaired subjective health and psychological wellbeing, providing a summary measure of overall subjective health status. The use of a composite measure allowed us to examine whether the combined deterioration across these domains is associated with IHD. In our study, we evaluated the association of self-rated health and the WHO 5 WellBeing Index with the risk of IHD. We found that good self-rated health and good mental wellbeing, compared to poor and average self-rated health and poor mental wellbeing, were associated with significantly lower odds of IHD in both the univariate binary logistic regression model (OR = 0.526, 95% CI 0.40–0.70) and the multivariate model that included other sociodemographic, lifestyle, and biological risk factors (OR = 0.692; 95% CI 0.22–0.93). Similar results we found for individuals having one of those factors: good self-rated health or good mental wellbeing (OR = 0.605 (95% CI 0.46–0.79) and OR = 0.737 (95% CI 0.56–0.97), respectively). Our results are consistent with previous reports of poor self-rated health and mental wellbeing index in individuals not only with CVD but also with other chronic diseases or conditions, such as mental, neurological, gastrointestinal, and other (37, 40, 41). A key distinction of our study from others is the use of a pooled variable combining self-rated health and mental wellbeing in the logistic regression models. In comparison, previous studies have either analysed only one of these variables or included both in separate models (42, 43). In our opinion, using such a pooled variable more effectively reveals the association with IHD compared to using the two variables separately in logistic regression models.

We also evaluated the association of lifestyle habits with the risk of IHD. Among all analysed lifestyle factors, only more frequent consumption of meat products was positively associated with the risk of IHD (OR = 1.308, 95% CI 1.02–1.38). Several overlapping pathways mediate the effects of diet, especially frequent consumption of meat products, on IHD: modulation of circulating lipids (especially LDL-cholesterol), systemic and vascular inflammation, endothelial function and oxidative stress, and interactions with the gut microbiome (e.g., trimethylamine *N*-oxide TMAO). Processed meats, higher in saturated fatty acids and trans fats, raise LDL and other atherogenic lipids, increasing the risk of changes in plaque formation (44). Gut microbes metabolize certain animal-derived nutrients (choline, l-carnitine) to TMAO, which has been linked experimentally and epidemiologically to atherosclerosis (45). This offers one of the plausible pathways linking some animal products to IHD.

Our study also identified influencing factors that were associated with good self-rated health and/or good mental wellbeing. Results revealed that increasing age and more frequent consumption of meat products and potatoes were significantly associated with lower odds of good self-rated health and/or good mental wellbeing. These findings are consistent with previous studies that have also reported a negative association between age and self-rated health or mental wellbeing (32, 40, 46). Several biological mechanisms may underlie the association between consumption of processed meat products and poor mental wellbeing, including symptoms of depression. Higher consumption of meat and processed meat products increases dietary intake of saturated fats, salt, nitrites and various additives. This dietary pattern is associated with alterations in gut microbiota composition. Dysbiosis leads to decreased production of health-promoting microbial metabolites, such as short-chain fatty acids (SCFAs), and increased production of detrimental gut-derived metabolites, including lipopolysaccharides (LPS) and isovaleric acid (47, 48). Meats are high in l-carnitine, a compound that is metabolized by gut microbiota into trimethylamine (TMA), which is then converted in the liver into trimethylamine *N*-oxide (TMAO) (49). Elevated circulating TMAO levels are associated with increased systemic inflammation - an increased activity of pro-inflammatory cytokines (e.g., IL-6, TNF-*α*) and the activation of inflammatory pathways is observed (50). Health-promoting microbial metabolites, including SCFAs, also influence vagus nerve activity and central nervous system functioning, and some can reach the brain directly via circulation (47).

In addition, microbiota alterations impair the synthesis and availability of neurotransmitter precursors and brain neurotransmitters—such as dopamine, serotonin, and norepinephrine—and interfere with their signaling pathways and disrupt the GABA–glutamate balance, which further contributes to emotional dysregulation (48, 51). Reduced SCFA production is also associated with diminished anti-inflammatory protection and increased intestinal epithelial permeability (52). Greater intestinal permeability contributes to endotoxemia, elevated circulating pro-inflammatory factors (e.g., IL-6, TNF-*α*) and systemic low-grade inflammation. Systemic inflammation affects the brain through several pathways, including increased blood–brain barrier permeability, vagus nerve signaling, monocyte trafficking, and direct cytokine actions on neural tissue. Resulting neuroinflammation activates microglia and astrocytes, promotes additional cytokine release, and disrupts mood, motivation, and emotional regulation (53, 54). Diet-induced inflammation and changes in gut microbiota composition can stimulate HPA axis hyperactivity, which has been associated with increased cortisol secretion, emotional dysregulation, and vulnerability to depressive symptoms (55, 56).

In our findings, participants with higher physical activity in leisure time, longer sleep duration per night, and more frequent fresh vegetables, fruits, and fish consumption were associated with a significantly higher odds of good self-rated health and/or good mental wellbeing. The Taiwan Longitudinal Study on Ageing Survey Report (TLSA) data showed that the mean number of days vegetables and fruits are eaten per week was significantly higher among people with WHO-5 wellbeing index scores >50 than among those with scores ≤50 (43). It has been shown that vegetables and fruits, apart from other useful nutrients, contain significant amounts of fiber and polyphenols, which have anti-inflammatory, neuroprotective, and prebiotic properties, and are associated with lower levels of systemic inflammatory markers (57, 58). Elevated levels of inflammatory markers have been linked with an increased risk of lower wellbeing (59). Fish is the primary dietary source of the long-chain omega-3 polyunsaturated fatty acids eicosapentaenoic acid (EPA) and docosahexaenoic acid (DHA), which have been shown to reduce depressive symptoms in clinical trials. In a cross-sectional analysis from the PREDIMED-Plus trial involving 6,587 older adults, moderate fish consumption - particularly of fatty fish rich in EPA and DHA - was significantly associated with a lower lifetime prevalence of depression and reduced depressive symptom severity. As depression is a strong predictor of poor self-rated health and overall wellbeing, these findings suggest that moderate fish consumption may contribute to better subjective health status, possibly through anti-inflammatory effects, enhanced neurotransmitter function, and neuroprotective mechanisms (60–62). Beyond biological mechanisms, psychological and behavioral pathways play one of a central role in explaining how dietary patterns influence mental health outcomes. These processes operate through self-regulation, reward processing, cognitive and emotional functioning, stress coping, and health-behavior reinforcement loops (63). Stable energy levels improve motivation, social engagement, and resilience to stress, all of which contribute to better mental wellbeing (64). Also, diets rich in fiber, complex carbohydrates, and healthy fats maintain steady glucose availability, supporting sustained attention, lower irritability, and more stable moods (65). Fruits and vegetables provide a broad spectrum of essential vitamins and micronutrients (etc. vitamin C, zink, magnesium) (66) which are essential for neurotransmitters synthesis, normal mitochondrial functioning and normal immune system functioning (67). All of these functions are critical for mood regulation and cognitive performance.

A cross-sectional study conducted in Japan also reported a significant association of certain dietary habits and physical activity with optimal wellbeing (the top quartile of the WHO-5 mental wellbeing index (scores 16–25)) (68). The odds of optimal wellbeing were significantly higher in people having over 30 min of intensive exercise more than twice a week and those having 1 h of walking each day, as compared to persons not exercising and walking, even after adjustment for age, sex, BMI, and sleep quality (OR = 1.58 and 1.29, respectively). It was indicated that there exists a bidirectional association between physical activity and life satisfaction and mental wellbeing. Physical exercise improves both physical and mental health and promotes psychological wellbeing. People with higher levels of wellbeing are usually more frequently physically active (69). Various biological mechanisms help explain the relationship between physical activity and wellbeing. One of them involves the stimulation of neurochemical changes in the brain by physical activity, which leads to the release of endorphins and an increase in the secretion of serotonin and dopamine (70, 71). The endorphins interact with the receptors in the brain, and the result of such interaction is higher levels of relaxation and lower stress (72). These changes are related to higher general wellbeing. Another mechanism concerns the regulation of cortisol, a stress-related hormone; physical exercise can moderate cortisol release, thereby reducing the negative impact of stress on mental health (73).

We did not find significant associations of good self-rated health and/or good mental wellbeing between smokers and non-smokers. Similarly, most studies using the WHO-5 as a tool for measuring mental wellbeing have also reported no notable differences in smoking habits between individuals with poor and good wellbeing (68, 74, 75). In contrast, other researchers have found differences in smoking habits between individuals with poor and those with good or excellent self-rated health (30, 42, 76). For instance, Orimoloye et al. reported a significantly higher proportion of regular smokers among individuals who rated their health as poor or fair (42). Conversely, Lee et al. and Dong et al. found a higher prevalence of regular smoking among those who rated their health as good or excellent (30, 76).

Based on the results of the present study, promoting good self-rated health and mental wellbeing appears to be a promising area for the prevention of IHD and overall health promotion. Equally important is addressing the factors that increase the likelihood of good self-rated health and mental wellbeing, such as encouraging regular physical activity and supporting healthy dietary habits among the population.

This study has several limitations. First, the cross-sectional design of our study limits the interpretation of findings, as it does not allow for the evaluation of causal relationships between self-rated health, mental wellbeing, and IHD. Second, we did not assess self-rated health, mental wellbeing, or the prevalence of IHD among individuals younger than 25 or older than 69 years. Including participants under 25 years could have lowered the overall participation rate; besides, in this age group, the prevalence of IHD, poor self-rated health, and poor mental wellbeing is typically low (77). In contrast, including individuals over 69 years would have increased the proportion of older adults participants - a group with a higher prevalence of chronic diseases and greater risk of reduced mental wellbeing and poor self-rated health, making them particularly interesting from a research perspective. However, due to financial constraints and the technical capacity of the study team, we limited the study sample to individuals aged 25–69 years. Third, although we adjusted for a quite large and relevant number of covariates in the logistic regression models, the unmeasured confounding factors may still have influenced the observed associations between self-rated health, mental wellbeing, and prevalence of IHD. Fourth, the random sample from the Lithuanian Population Register is representative of the Kaunas population by gender and age, and its size is adequate for assessing health indicators. As a randomly selected urban sample, the findings of our study may be partially generalizable to the Lithuanian population as a whole. Despite such limitations, the strengths of our study include, first, the use of data collected from a sample that was randomly selected from the general population. Second, we applied the WHO-5 mental wellbeing evaluation tool, which has been previously standardized, validated (14), and the latter has been translated into the Lithuanian language. Third, to the best of our knowledge, this is the first study to use a pooled variable combining self-rated health and mental wellbeing in logistic regression models to analyze the odds of IHD.

## Conclusion

This study highlights the significant association of good self-rated health and mental wellbeing status with a lower odds of IHD among adults aged 25–69, even after adjusting for the main risk factors. Furthermore, specific lifestyle habits - including regular physical activity, sufficient sleep, and healthy nutrition habits (rich in fruits, vegetables, and fish) - were positively associated with better self-rated health and mental wellbeing. However, more frequent consumption of meat products and potatoes is associated with a lower odds of good self-rated health and mental wellbeing. These findings underscore the importance of promoting healthy lifestyle behaviours and mental wellbeing as part of comprehensive CVD prevention strategies.

## Data Availability

The raw data supporting the conclusions of this article will be made available by the authors, without undue reservation.
